# Retraction: Gel scaffolds and emerging applications in biomedicine

**DOI:** 10.1039/d3ra90081a

**Published:** 2023-09-04

**Authors:** Mani Rajasekar, Manivannan Lavanya

**Affiliations:** a Centre for Molecular and Nanomedical Sciences, International Research Centre, Sathyabama Institute of Science and Technology (Deemed to be University) Chennai – 600 119 Tamilnadu India drmrajasekar.irc@sathyabama.ac.in mrajasekar_83@yahoo.com +91-44-24503814 +91-9710230530

## Abstract

Retraction of ‘Gel scaffolds and emerging applications in biomedicine’ by Mani Rajasekar *et al.*, *RSC Adv.*, 2022, **12**, 15925–15949, https://doi.org/10.1039/D2RA00924B.

The Royal Society of Chemistry, with the agreement of the authors, hereby wholly retracts this *RSC Advances* article due to a significant amount of text overlap with a number of different sources in Sections 2–6 and the Conclusions of this review article. Although the sources have been cited in this article, it was not made clear that the text was reproduced from the articles.

Signed: M. Rajasekar, M. Lavanya

Date: 16th August 2023

Retraction endorsed by Laura Fisher, Executive Editor, *RSC Advances*

## Supplementary Material

